# A Salutary Role of Reactive Oxygen Species in Intercellular Tunnel-Mediated Communication

**DOI:** 10.3389/fcell.2018.00002

**Published:** 2018-02-06

**Authors:** Dacheng Liang

**Affiliations:** ^1^Hubei Collaborative Innovation Center for Grain Industry, School of Agriculture, Yangtze University, Jingzhou, China; ^2^Engineering Research Center of Ecology and Agricultural Use of Wetland, Ministry of Education, Yangtze University, Jingzhou, China

**Keywords:** ROS, intercellular movement, interorganelle transport, membrane protrusions, plasmodesmata, tunneling nanotubes (TNTs), macromolecule movement, multicellularization

## Abstract

The reactive oxygen species, generally labeled toxic due to high reactivity without target specificity, are gradually uncovered as signaling molecules involved in a myriad of biological processes. But one important feature of ROS roles in macromolecule movement has not caught attention until recent studies with technique advance and design elegance have shed lights on ROS signaling for intercellular and interorganelle communication. This review begins with the discussions of genetic and chemical studies on the regulation of symplastic dye movement through intercellular tunnels in plants (plasmodesmata), and focuses on the ROS regulatory mechanisms concerning macromolecule movement including small RNA-mediated gene silencing movement and protein shuttling between cells. Given the premise that intercellular tunnels (bridges) in mammalian cells are the key physical structures to sustain intercellular communication, movement of macromolecules and signals is efficiently facilitated by ROS-induced membrane protrusions formation, which is analogously applied to the interorganelle communication in plant cells. Although ROS regulatory differences between plant and mammalian cells exist, the basis for ROS-triggered conduit formation underlies a unifying conservative theme in multicellular organisms. These mechanisms may represent the evolutionary advances that have enabled multicellularity to gain the ability to generate and utilize ROS to govern material exchanges between individual cells in oxygenated environment.

## Introduction

Various cellular metabolic processes, ranging from energy capture and storage in chloroplast, long chain carbon metabolism in peroxisome, to energy generation in mitochondria, all under normal physiological condition, accompany with the generation of one type of products: the reactive oxygen species (ROS) (Halliwell and Gutteridge, 1989; Scandalios, 1997; Davies, 2000; Apel and Hirt, 2004). The mechanisms of ROS formation during these metabolic processes abound (Hinshaw et al., 1986; Al-Mohanna and Hallett, 1987; Quinn et al., 1989; Apel and Hirt, 2004; Asada, 2006; Pospíšil, 2009). The most well-known is oxygen's stepwise reduction principle in which oxygen molecule in its ground state can only accept one electron at a time to be reduced into superoxide anion (${\text{O}}_{2}^{•}-$), then further into hydrogen peroxide (H_2_O_2_), hydroxyl radical (·OH) and water, or excited into singlet oxygen (^1^O_2_) via energy transfer (Klotz, 2002; Turrens, 2003; Apel and Hirt, 2004; Sharma et al., 2012). These ROS intermediates energetically react with biomolecules in a non-selective way and perhaps prompt further production of other destructive radicals, thus posing great threats to cellular integrity and viability. Coping with aerobic metabolism, aerobic life has evolved anti-ROS system including non-enzymatic antioxidant and ROS-scavenging enzymes to ameliorate the toxic effects imposed by ROS (Raghu et al., 1986; Abid et al., 2000). Actually, the tug of war between ROS and antioxidant system has been on play since evolution of early life living in less oxic environment (Moldovan et al., 2000; Harfouche et al., 2005); for instance, the advent of enzymatic antioxidants such as superoxide dismutase (SOD), catalase and peroxiredoxin in organisms appearing on Earth around 4.1–3.5 billion years ago—around 1 billion years earlier than the great oxidation event, which highlights the paramount role of anti-ROS system in life evolution on Earth. This can be further seen from photosynthetic organisms that are equipped with a host of delicate, sophisticated non-enzymatic antioxidants as diverse as vitamins (e.g., Vitamin C, Vitamin E), amino acids (e.g., proline), peptide (e.g., GSH), carotenoids (e.g., β-carotene, lycopene, lutein), flavonoids (e.g., quercetin, catechin), and hormone (e.g., melatonin) (Sundaresan et al., 1995; Choi et al., 2005; Connor et al., 2007; Schröder et al., 2007; Lee et al., 2009).

Existence of such colossal antioxidants in aerobic life may easily lead to an illusion that ROS is flat-out toxic and doing nothing good. On the contrary, the past several decades have seen the growing evidence of ROS as important signaling molecules. The first recognized biological value of ROS in plants came from their integration into defense system against pathogen. Earlier experiments found hydrogen peroxide served as important signal to trigger local cell death and to induce antioxidant activation in adjacent cells (Daszkowska-Golec and Szarejko, 2013) and systemic acquired resistance (Shimazaki et al., 2007; Song et al., 2014). Derived from oxidative burst, one of the earliest events following pathogen challenge, H_2_O_2_ can rapidly—within several minutes—accumulate to a considerable level, e.g., magnitude of millimolar concentration in soybean cells (Apostol et al., 1989; Stahl and Simon, 2013). An analogous scenario is found in a confined organelle, or phagosome of immune cells, where ROS, produced at the millimolar quantities of H_2_O_2_, are used to kill intruding pathogens (Babior et al., 1973; Lucas and Lee, 2004; Adamec, 2006, 2007; Roelfsema and Hedrich, 2010). Later more than a decade studies revealed that the intentional production of ROS was largely driven by the activity of NOX complex (NADPH oxidase) (Royer-Pokora et al., 1986; Teahan et al., 1987; Babior, 2004; Bedard and Krause, 2007) or its plant counterpart, RBOH-NADPH oxidase (Torres et al., 2002; Sagi and Fluhr, 2006; Suzuki et al., 2011). Not surprisingly, other enzymes characterized from both animals and plants, such as peroxidase (Klebanoff, 1970; Bindschedler et al., 2006; Choi et al., 2007; O'Brien et al., 2012; Kimura et al., 2014), oxidoreductase (e.g., xanthine oxidase, Hille and Nishino, 1995; Harrison, 2002; Zarepour et al., 2010; Ma et al., 2016), oxalate oxidase (Requena and Bornemann, 1999), and oxygenase (e.g., cyclooxygenase and lipoxygenase) also contribute to the internal ROS production.

In addition to the involvement in biotic defense, these ROS-generating machineries together with antioxidants act as essential modulators of redox signaling pathways capable of effecting changes in a plethora of biological processes including, not limited to, abiotic stress, cell growth and programmed cell death, cell proliferation, and cell differentiation. For more excellent reviews, reader is referred to those by Holmström and Finkel (2014), Río and Puppo (2009), Rhee (2006), Finkel (2011), Farmer and Mueller (2013), Foyer and Noctor (2005), Mittler et al. (2011), Ray et al. (2012), Baxter et al. (2014), and Sewelam et al. (2016). Nevertheless, a latent aspect in the repertoire of ROS function has not yet brought into discussion until recently; their emerging role in modulating cell-to-cell information flow through plasmodesmata (PD) in plant and their inductive capability to produce membrane protrusions between mammalian cells, as well as among organelles in plant cells. Thanks to some new approaches adopted to uncover this long-neglected regulatory mechanism, we can get the glimpse of how ROS, particularly H_2_O_2_, are generated and incorporated into intercellular bridge-mediated communication by promoting membrane protrusions formation (in both animal and plant cells) and cell wall remodeling (in plant cells). This review is focused on the genetic control of intercellular movement of various molecules including symplastic dye, small RNA, and proteins, and outlines the underlying mechanisms that are shared across plant and animal kingdoms.

## Dyeing symplastic movement—role for ROS

Intercellular transport in plants is believed to be one of the most fundamental processes for which PD embedded in the cell wall but connecting cytoplasm of neighbor cells is essential to enable the cell-cell symplastic exchange. The PD-mediated intercellular communication can be observed through symplastic dye loading experiment, e.g., CFDA-coupling (Wang and Fisher, 1994), Lucifer Yellow CH injection (van Kesteren et al., 1988), and HPTS loading experiment (Gisel et al., 1999). Copious data accumulated from studies on symplastic tracer loading in model plant *Arabidopsis*, as well as in monocots and woody plants, provides indicative insights into the recognition of relative disconnection from surrounding tissues in some types of cell clusters/tissues, or namely the symplastic domain (van der Schoot and van Bel, 1990; Botha and van Bel, 1992; Rinne and van der Schoot, 1998; Ding et al., 2003) in which the cell-to-cell connection is highly united into a continuity, but temperally and spacially cut-off from other cells/tissues. This phenomenon posed an immediate question as how the symplastic domains are formed and how they are regulated during plant development.

Genetic screens from the embryo-defective collection, which was predicated on the premise that defective cell-to-cell transport would result in the embryo abnormality or growth arrest, recovered mutants showing increased plasmodesmatal conductance of symplastic dye, e.g., the 10-kD F-dextran (Kim I. et al., 2002; Kobayashi et al., 2007; Burch-Smith and Zambryski, 2010), or decreased movement of small dye, e.g., HPTS (Xu et al., 2012), at different stages of embryo development, further adding weight to the idea that the symplastic domain is dynamically regulated (Gisel et al., 1999; Ding et al., 2003). However, subsequent outcome from map-based cloning of causal mutation in these mutant lines attested to a diversified genetic pathway in which the genetic component seems not directly link to each other, either spacially or chemically. For instance, both *ISE1* and *ISE2* (*INCREASED SIZE EXCLUSION LIMIT OF PLASMODESMATA 1* or *2*) gene encode two different RNA helicases that are involved in the very basic aspect of RNA metabolism ranging from RNA transcription, splicing to degradation, and that are localized to mitochondria and chloroplast respectively, whereas *DSE1* (*decreased size exclusion limit 1*) gene encodes a WD-repeat protein that is distributed in both cytoplasm and nucleus. Although these genes controlling the cell-to-cell movement of symplastic tracer appear to have no apparent intrinsic coherence in light of genetic and/or biochemical pathways or even the spacial occurrence relating to PD, TEM dissection has shown these mutants have altered architecture of PD; with increased frequency of twinned and branched PD in increased tracer movement mutant or decreased frequency of twinned and branched PD in decreased tracer movement mutant.

These results have clearly shown PD-mediated transport indicated by symplastic tracer is paramount, and as such the mutants are often displaying severe phenotypic abnormalities as diverse as retarded growth, altered flowering time, dysfunctional reproductive organs, and even embryo lethal from strong mutant allele. Concomitant to the abnormal growth of mutant lines, the ROS level is apparently higher in the *ise1* mutant as opposed to the wild type plant, which might be explained as a result of developmental disruption of mitochondria or chloroplast (Burch-Smith et al., 2011). Though ROS measurement is not available to *dse1* or *ise2* mutant, anthocyanin, known as stress-inducible phytochemical, is accumulated in the embryo of *dse2* mutant and another allele *tan* mutant (Xu et al., 2012), and cotyledon of *ise2* mutant as well (Kobayashi et al., 2007), tentatively suggesting that PD-mediated transport might be subject to ROS-related regulatory mechanisms.

**Figure 1:**
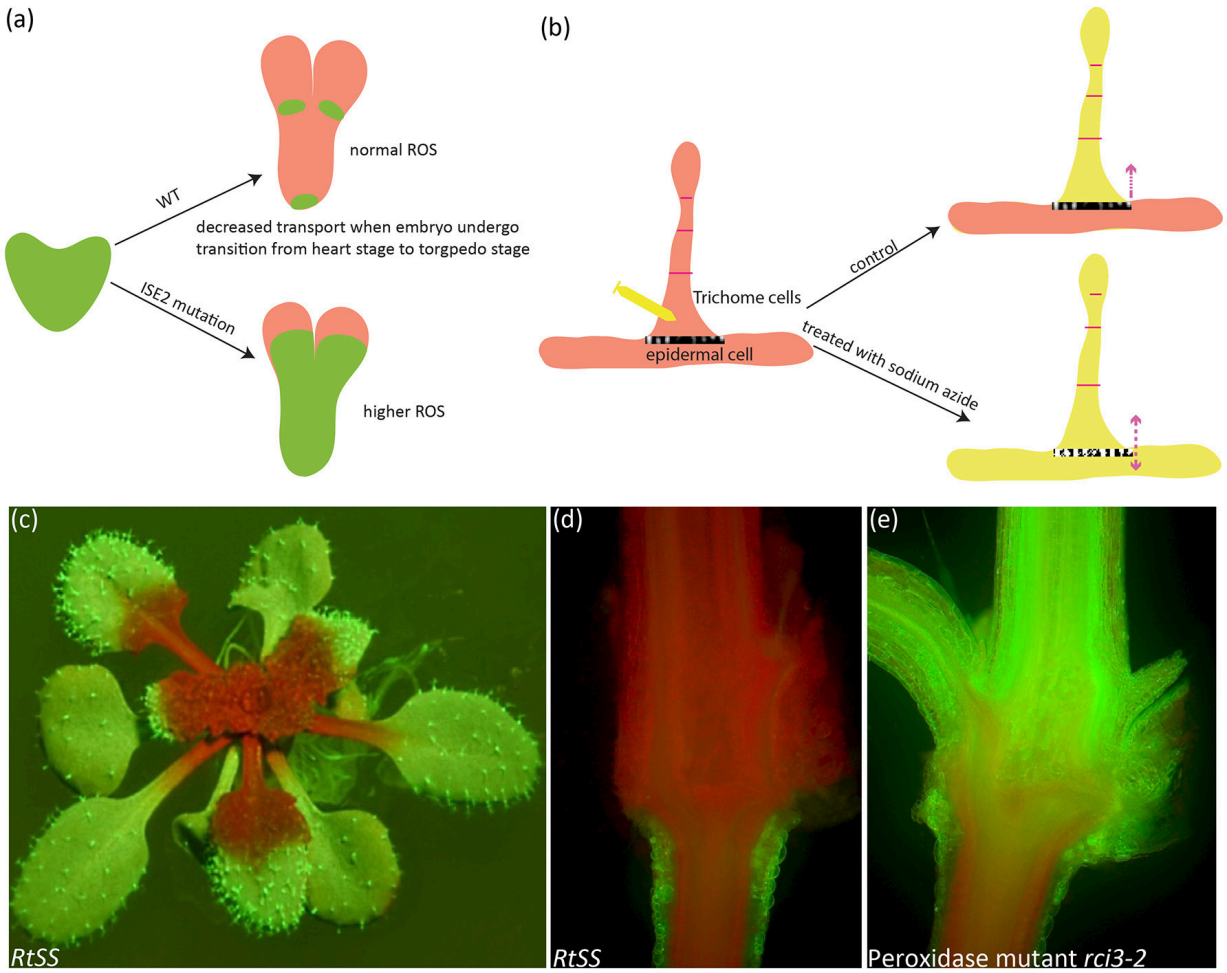
Symplastic dye movement is associated with ROS. The symplastic dye 10 kd F-Dextran moves freely at the heart stage of WT and *ise2* mutant, but its movement is restricted at the torpedo stage due to the formation of symplastic domain. Higher ROS content in the *ise2* mutant lifts this restriction. Green depicts F-Dextran **(a)**. In the epidermal/trichome (e/t) boundary, LYHC dye moves freely between the trichome cells, but cannot cross the e/t boundary into epidermal cell. Sodium azide, a known ROS-inducing agent can break this blockage. Yellow color depicts the LYCH. **(c–e)** A root-to-shoot silencing system (RtSS) demonstrates the long-distance movement of small RNA-mediated gene silencing movement. **(c)** An RtSS plant shows the silencing pattern after 15-day Dex induction. **(d,e)** Longitudinal sectioning of RtSS has shown that silencing front moves from the root to the shoot **(d)**, but the mutation in type III peroxidase RCI3 (R145K) heavily retards the movement **(e)**. **(a,b)** were drawn according to the description by Kim I. et al. (2002) and Christensen et al. (2009) respectively. **(e)** was reprinted from Liang et al. (2014) under a Creative Commons Attribution License granted to Peer J.

Experiments with microinjection showed sodium azide, an agent known to inhibit electron transport in mitochondria complex IV (Petersen, 1977; Yoshikawa et al., 1998), thus leading to ROS augmentation in mammalian cells (Duranteau et al., 1998) and also mitochondrial oxidation in *Arabidopsis* (Schwarzländer et al., 2009), can enlarge PD pore and enhance the cell-to-cell movement of various dyes (Tucker, 1993). Moreover, the size exclusion limit (SEL) of cortical PD in wheat roots under oxidative stress induced by azide can be increased from < 1 KD to between 5 and 10 kD (Cleland et al., 1994). Astonishingly, this oxidative effect was further applied to breaking down the epidermis-trichome symplastic boundary by increasing SEL in tobacco leaf (Figure 1) (Christensen et al., 2009).

Genetic studies on PD-mediated tracer movement have provided qualitative perspective on cell-cell transport and lent much credence to the test of mathematically modeled permeability. Rutschow et al. (2011) quantified the intercellular permeability of *Arabidopsis* root meristematic regions by applying the diffusion model to CF bleaching experiment with confocal microscope, and generated a new measurement of PD conductivity, which is nearly an order of magnitude larger than the reported with dye microinjection (Goodwin et al., 1990) (Table 1). Nevertheless, this value discrepancy may lie with the responsiveness of PD when subjected to the perturbation of handling or environmental stimuli, or the variation of PD components and structure in different tissues/organs (Radford and White, 2001; Roberts and Oparka, 2003). The validity of this model was further corroborated in two *Arabidopsis* lines in which the PD-mediated transport is in defect shown before. With this confirmation, the authors made a straightforward test on the role of H_2_O_2_ in intercellular transport that was contrarily demonstrated to be in association with PD conductance in two ROS-elevated mutants: *ise1* mutant with increased transport (Stonebloom et al., 2009), but *gat1* with decreased transport (Benitez-Alfonso et al., 2009). At relative low concentration of 0.6 mM, H_2_O_2_ can double the PD permeability, but the opposite effect was observed when plants were exposed to higher concentration, say, 6 mM (Rutschow et al., 2011), alluding that the extent to which the inner ROS accumulated in these mutants (Benitez-Alfonso et al., 2009; Stonebloom et al., 2009), or alternatively in which subcellular organs (Stonebloom et al., 2012), may explain the contradictory repercussions observed in two independent genetic studies. Further studies would aim at defining the boundary of H_2_O_2_ concentration and exploring the cause of adverse effect when plants are exposed to H_2_O_2_.

**Table 1:** Movement rate of various substances in plants.

**Mobile substances**		**Movement rate or time cost**	**Type of movement**	**Cell/Tissue location**	**Direction**	**Species**	**Reference**
Artificial dye	CF	4.0 mm/h	Cell-to-cell	Epidermal PD, leaf	Radial diffusion	Egeria densa	Goodwin et al., 1990
	FITC-Glu	0.56 mm/h					
	FITC-(Glu)_2_	0.41 mm/h					
	FITC-(Gly)_6_	2.4 x 10^−2^ mm/h					
	FLGGL	3.24 x 10^−4^ mm/h					
	CF	≈31 mm/h	Cell-to-cell	Close to QC in RAM	Longitudinal	Arabidopsis	Rutschow et al., 2011
	CF with H_2_O_2_	94 mm/h					
Photoassimilate and water	^14^C	430 mm/h	Long-distance	Petiole, stem	Downward	Nicotiana glutinosa	Helms and Wardlaw, 1978
	^14^C	500–1,350 mm/h	Long-distance	Leaf blade, midrib, petioles	Downward	Sugar beet	Mortimer, 1965
	water	900–1,440 mm/h	Long-distance	Stem	Downward	Tobacco, tomato, castor bean and poplar	Windt et al., 2006
	water	900 mm/h	Long-distance	Hypocotyl	Downward	Castor bean	Peuke et al., 2001
Virus	TMV	2.5 x 10^−2^ mm/h	Cell-to-cell	Leaf	Radial	Nicotiana benthamiana	Boyko et al., 2000
	TMV	3.8 x 10^−2^ mm/h	Cell-to-cell	Leaf	Radial	Tobacco	Kawakami et al., 2004
	TAMV, TMV and PVX	80 mm/h	Long-distance	Stem	Both upward and downward	Tomato	Capook, 1949
	TMV	1.1–23.3 mm/h	Long-distance	Petiole, stem	Both upward and downward	Nicotiana glutinosa	Helms and Wardlaw, 1978
Mobile Silencing	Root-to-shoot mobile silencing	1.6 x 10^−2^ mm/h	Cell-to-cell and long-distance	Hypocotyl	Shootward	Arabidopsis	Liang et al., 2012
		2.3 x 10^−3^ mm/h		HEJ			
	Systemic silencing	20–34 days	Long-distance	Leaf, stem	Upward	Nicotiana benthamiana	Voinnet et al., 1998
	Systemic silencing	21–28 days	Long-distance	Leaf, stem	Both upward and downward	Tobacco	Crete et al., 2001
	Systemic silencing	21 days	Long-distance	Leaf, stem	Upward	Tobacco	Palauqui et al., 1997

*CF, Carboxyfluorescein; FITC, fluorescein-isothiocyanate isomer I; FGlu, fluorescein glutamic acid; F(Glu)_2_, fluorescein glutamyl-glutamic acid; F(GIy)_6_, fluorescein hexaglycin; FLGGL, fluorescein leucyl diglutamyl-leucine; QC, Quiet Center; RAM, root apical meristem; TMV, tobacco mosaic virus; TAMV, Tomato aucuba mosaic virus; PVX, potato virus X; HEJ, Hypocotyl-epicotyl junction*.

## Movement of small RNA-mediated post-transcriptional gene silencing is controlled by hydrogen peroxide

RNA silencing is a conserved defense system against foreign nucleic acid species across a wide range of organisms. Interestingly, this process involving small RNA in size of 21–24 nt is not contained *in situ* but rather elicits a non-cell-autonomous transmission—in a mode of either short distance movement or of long-distance movement, or both (Kalantidis et al., 2008; Liang et al., 2011; Melnyk et al., 2011). One crucial question concerning the short/long-distance mobile silencing is how the movement of silencing signal is subject to intercellular traffic control.

In *C. elegans*, intercellular transport of silencing signals depends on the activity of a double stranded RNA (dsRNA) transporter, or SID-1 which enables transport of the length of 50 bp up to 500 bp dsRNAs (Winston et al., 2002). It seems no counterpart of SID-1 in plants is responsible for the silencing movement. Thus, what factors or mechanisms in plants are employed to render silencing dissemination has been very intriguing subject of signal transmission. Long-sought efforts of genetic screening based on the short-range silencing movement represented by, e.g., Atsuc2-hp*PDS* or Atsuc2-hp*Sul*, in an intention to identify cellular components constituting either cytoskeletal features or cell channels result in the identification of genetic factors that are mainly constituting the silencing machinery or silencing pathways (Melnyk et al., 2011). Besides, the time the silencing itself takes to travel through (assumed in the phloem system) is surprisingly much longer than photoassimilates in the phloem sap (Table 1). These situations leave the traffic control of mobile silencing signal through PD unsettled, and also translate into a need to tackle the issues around the way the silencing movement may take.

In an effort to mimic the grafting system, Liang et al. (2012) established a root-to-shoot long-distance mobile silencing system (RtSS) in which the initial site of silencing signal generation is spaciously separated from the signal receiving tissue (Liang et al., 2012), thus enabling to follow the route, monitor the time, and tease out the details for an upward systemic silencing. Indeed, a reiterated cell-to-cell silencing movement was observed making up the upward long-distance silencing. As discussed in previous section, the symplastic domain validated by symplastic dye makes its own hurdle on the movement of this small RNA-mediated gene silencing, resulting in the variation of movement rate during the course of systemic spreading (Table 1); for example, the slower movement in the hypocotyl-epicotyl junction than in the hypocotyl.

**Figure 2:**
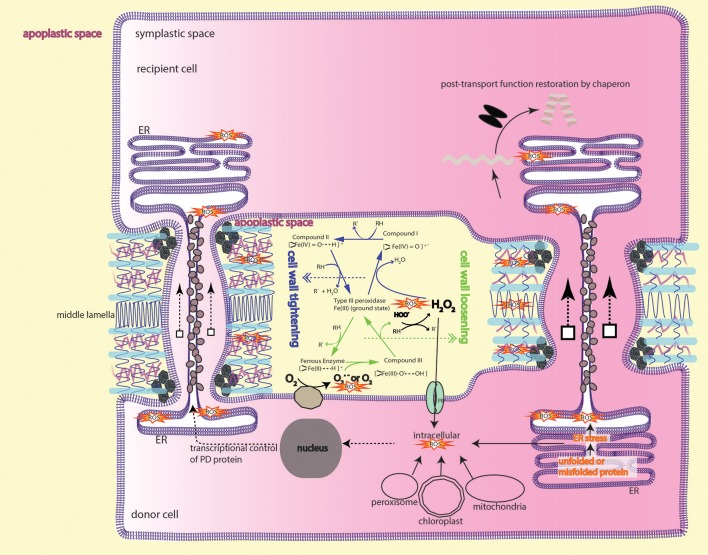
Peroxidase-catalyzed cell wall remodeling dictates PD transport. In the apoplastic space (light yellow), type III peroxidase (POX) performs peroxidative cycle (blue arrows) and oxidative cycle (green arrows) to regulate H_2_O_2_ and superoxide/O2 level. Apoplastic ROS from the two cycles lead to cell wall tightening or loosening by crosslinking or depolymerizing cell wall components, thereby shrinking or enlarging the plasmodesmatal passage. Apoplastic H_2_O_2_ can also, through aquaporins-mediated transmembrane transport, enter the cytoplasm to initiate downstream signaling events, e.g., MAPK cascade signaling and cytoskeletal remodeling, resulting in membranous protrusion (see the section ROS is required to form intercellular/interorganelle bridge for the details). ROS imbalance inflicted by mutation in ISE1, ISE2, GAT1 localized in the subcellular compartments also impacts on intercellular transport. It's currently unknown whether or how ROS from subcellular compartments contribute to apoplastic ROS pool. Protein that moves through PD sleeve may be subject to ER stress-induced ROS, thus requires a post-movement refolding (details in the text). The peroxidase/oxidase cycling was adapted from Berglund et al. (2002) with the permission from Rightslink®.

Forward genetic screening on RtSS, aiming to understand movement control imposed by PD and/or symplastic domain, identified *tsg1* (Tiao-shan-gong 1, meaning in Chinese the mountain porter who facilitates transport over a mountain) mutant that shows only deficiency in the silencing movement while maintaining the production of silencing signal (Liang et al., 2014). Genetic and molecular studies aided by genome re-sequencing revealed that *tsg1* is an allele of *rci3* gene which encodes type III peroxidase (POX) enzyme. An Arg to Lys substitution in a highly conserved motif in the RCI3 peroxidase resulted in the reduced hydrogen peroxide level in the mutant line, in agreement with the function of POX which can regulate H_2_O_2_ level through peroxidative and oxidative cycle (Figure 2) (Berglund et al., 2002; Passardi et al., 2004b). Most remarkably, direct replenishment of H_2_O_2_ to plant growth medium not only can restore silencing movement in the mutant, but also augment the rate of silencing movement in RtSS system (Liang et al., 2014) and the range of vascular silencing (Liang et al., 2014) in JAP3 line (Smith et al., 2007). Conversely, depletion of H_2_O_2_ in plant growth medium by Catalase or MnO_2_ catalyst can simply and efficiently reduce the silencing movement in both RtSS and JAP3 line (Liang et al., 2014), unambiguously pinning down the role of H_2_O_2_ in small RNA-mediated gene silencing movement.

The silencing front moves through hypocotyl to shoot in a way of cell-to-cell transmission, and spreads—analogously to the PD-mediated symplastic dye movement—with considerable resistance into symplastic domains, such as hypocotyl epidermis (Duckett et al., 1994) and the hypocotyl-epicotyl junction (Liang et al., 2012). Thus, it's legitimate to assume that the cell-to-cell channel or PD is the route for silencing RNAs migration. Immunogold-labeling assay and Cerium(III) cytochemical TEM (Transmission electron microscopy) assay respectively localized the POX and H_2_O_2_ to PD vicinity and cambial cell wall in the form of punctate aggregates associated with PD (Ehlers and van Bel, 2010), suggesting the role of H_2_O_2_ and/or ·OH in PD-associated process. Earlier insights revealed by many biochemical studies that elevation or decreasing of H_2_O_2_ resulted in cell wall loosening or stiffening (Fry, 1998; Schweikert et al., 2000; Schopfer, 2001; Liszkay et al., 2004; Müller et al., 2009; Kunieda et al., 2013) led the authors to propose a new model in which the PD-mediated transport is modulated through cell wall remodeling (Liang et al., 2014). In this model, ROS molecules like H_2_O_2_, ·OH are working as biochemical scissor, cleaving cell wall polymers, altering the cell-wall networks, and inevitably changing the passage of PD imbedded in the cell wall (Figure 2). This model emphasizes the role of ROS in apoplastic space and, for the sake of simplicity, has not considered the ROS signaling happening in the symplastic domain due to lack of experimental data. Thus, further investigation regarding to the intracellular aspects of ROS-initiated cascade (Mangano et al., 2016; Schmidt et al., 2016; Kimura et al., 2017) will reveal novel insights into the interaction between symplastic and apoplastic ROS signaling pathways.

## Protein diffusion is associated with ROS

**Figure 3:**
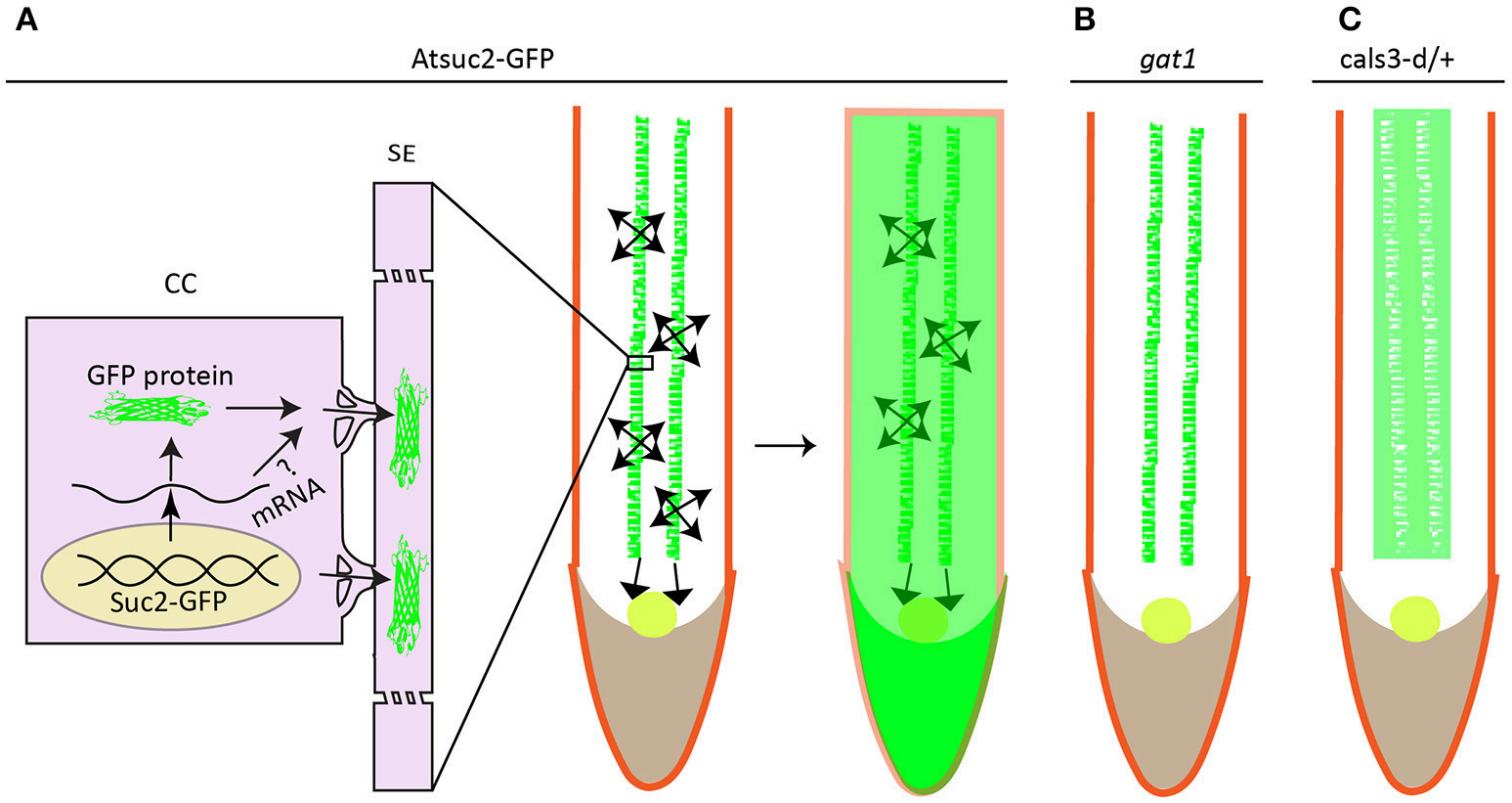
Protein unloading from phloem to surrounding cells in root is associated with redox homeostasis. GFP is specifically expressed in the companion cell under the control of AtSuc2 promoter. It's not retained in the companion cell but rather disperses into sieve element (SE) and other parts of roots including stele, cortex, RAM (round circle), and root cap **(A)**. mRNA of GFP is less likely to move into surrounding cells to be translated into fluorescence signal as plants harboring either bigger size of GFP or subcellular targeted GFP only show green fluorescence in the companion cells. Loss-of-function mutation in thioredoxin gene *GAT1* for redox regulation **(B)**, or gain-of-function mutation in callose synthase gene *Cals3* shows defective GFP trafficking **(C)**. **(A–C)** were drawn according to the description by Imlau et al. (1999), Benitez-Alfonso et al. (2009), Vatén et al. (2011) respectively.

It is generally established that specification of cell types through cell proliferation and differentiation largely depends on positional cues (Sessions and Yanofsky, 2001; Van Norman et al., 2011), which are hypothesized to exert their non-cell autonomous effects from a certain distance. Plant meristem-maintaining factors, such as WUSCHEL (WUS) (Yadav et al., 2011), KNOTTED-1 (KN1) (Lucas et al., 1995; Kim J. Y. et al., 2002), SHORT-ROOT (SHR) (Nakajima et al., 2001; Gallagher et al., 2004), TMO7 (Schlereth et al., 2010), are produced either locally or in a spatially discrete way, but can move in a short distance into neighboring cells to initiate downstream regulatory programs, thereby exemplifying the typical positional signaling mediated by mobile transcriptional factors (see reviews by Wu and Gallagher, 2012; Han et al., 2014; Long et al., 2015). The immediate question would be of interest to pinpoint the pathways whereby the mobile proteins are signaling their effects, e.g., the secretory pathway or PD pathway. Genetic screening on pSUC2::GFP system in which the distribution of GFP fluorescence is expanded from phloem into RAM due to a free GFP protein diffusion (Imlau et al., 1999) (Figure 3) identified gain-of-function mutation in *CALS3* gene whose product catalyzes the polymerization of β-1,3-linked D-glucose or callose formation (Vatén et al., 2011). Mutation in CALS3 gene not only congealed GFP diffusion, but also substantially retarded root growth. Presumably, the phenotypic change in CALS3 gain-of-function mutant resulted partially, at least, from the impaired movement of SHR protein and microRNA miR165 (Vatén et al., 2011; Wu et al., 2016), as the aperture of PD was decreased by increased callose deposition in the PD neck of mutant, which is exactly consistent with tremendous studies on the effect of callose on PD conductivity (see reviews by Roberts and Oparka, 2003; Chen and Kim, 2009; Zavaliev et al., 2011; Burch-Smith and Zambryski, 2012; De Storme and Geelen, 2014; Sager and Lee, 2014). Likewise, in the shoot tip, PD aperture reduction by CALS3m-directed callose deposition in the SAM restricts WUS protein movement from organizing center to stem cells residing in the outermost cell layers (Daum et al., 2014), leading to developmental defects similar to *wus* mutant. These studies have clearly assigned the rout for mobile transcriptional factor, and positional signaling through PD is indeed playing very important role in cell division and cell identity, thus tissue patterning (Otero et al., 2016).

An earlier independent EMS screening on pSUC2::GFP system with the same aim to uncover mutant showing reduced GFP traffic, pointed to a new regulatory pathway linked to redox homeostasis (Benitez-Alfonso et al., 2009). Benitez-Alfonso et al. demonstrated that GFP movement in the phloem-RAM interface ground to a halt in an epiallele of *gat1* gene encoding a plastid-localized m type thioredoxin (Figure 3), which essentially contributes to the regulation of chloroplast redox state (Vieira Dos Santos and Rey, 2006; Serrato et al., 2013; Nikkanen and Rintamäki, 2014; Buchanan, 2016). Indeed, examination on ROS level in this mutant detected an elevated oxidative staining with 3,3′-diaminobenzidine (DAB) compared to the wild type plant, apparently contradicting to these findings (Stonebloom et al., 2009; Rutschow et al., 2011; Liang et al., 2014) in which the increased ROS level are positively associated to PD-mediated transport. As ISE1, ISE2, GAT1 protein are localized in different organelles, one approach to reconcile this contradiction is to determine the sub-cellular redox state indicated by the redox-sensing green fluorescent protein fused with specific compartment-targeting signal; a higher oxidized state of mitochondria in the *ise1* mutant or in WT plants treated with Salicylhydroxamic acid (SH) and a reduced chloroplast redox in *ise2* are all positively correlated to plasmodesmal transport whereas oxidized state of chloroplast in paraquat-treated WT plants resulted in decreased plasmodesmal transport irrespective of the redox states of either mitochondria or cytoplasm (Stonebloom et al., 2012). The decreased PD transport in *gat1* mutant seems concordant with the proposed model (Stonebloom et al., 2012) as paraquat-treated pSUC2::GFP plants showed exactly the same phenotype to *gat1* mutant (Benitez-Alfonso et al., 2009). Comprehensive work such as direct comparison between mutants, e.g., the *ise1* (Stonebloom et al., 2009), *ise2* (Kobayashi et al., 2007), *gat1* (Benitez-Alfonso et al., 2009), *dse1* (Xu et al., 2012) with deficiency in PD transport, may be needed to construct a clearer picture of what effects the site of ROS production may inflict on PD conductance.

### Different ROS and their possible roles

The question of whether different ROS have different roles in regulating PD transport remains a mystery, but looms, indeed, large, which can be inspired from the findings that superoxide and hydrogen peroxide differently regulate cell proliferation, cell differentiation, and root growth in *Arabidopsis* (Dunand et al., 2007; Tsukagoshi et al., 2010; Müller et al., 2015). Tsukagoshi et al. (2010) reported a novel pathway involving three peroxidases (Per39, Per40, Per57) that function to balance the distribution of hydrogen peroxide and superoxide in the transition zone of cell proliferation and cell differentiation. Disturbance of ROS level by overexpression or disruption of peroxidase, can alter the ROS homeostasis in the transition zone, leading to increased or decreased root meristematic cell number. Interestingly, negative regulation of peroxidase expression requires a mobile transcriptional factor, UPBEAT1, which appears to move from root cap to elongation zone to repress peroxidase transcription and allows H_2_O_2_ accumulation in the differentiated zone. In the light of abovementioned evidence on the role of ROS in PD and recent evidence on the role of ROS in root development (Müller et al., 2015; Orman-Ligeza et al., 2016; Yu et al., 2016), it is compelling to speculate that developmental regulation via ROS signaling in root is, at least partially, coming from the oxidative/reductive impact on PD transport. However, this speculation should be tested, and particularly the nature of ROS in relation to cell types and PD function needs to be delineated.

### ROS stress, chaperon, and intercellular protein movement

**Figure 4:**
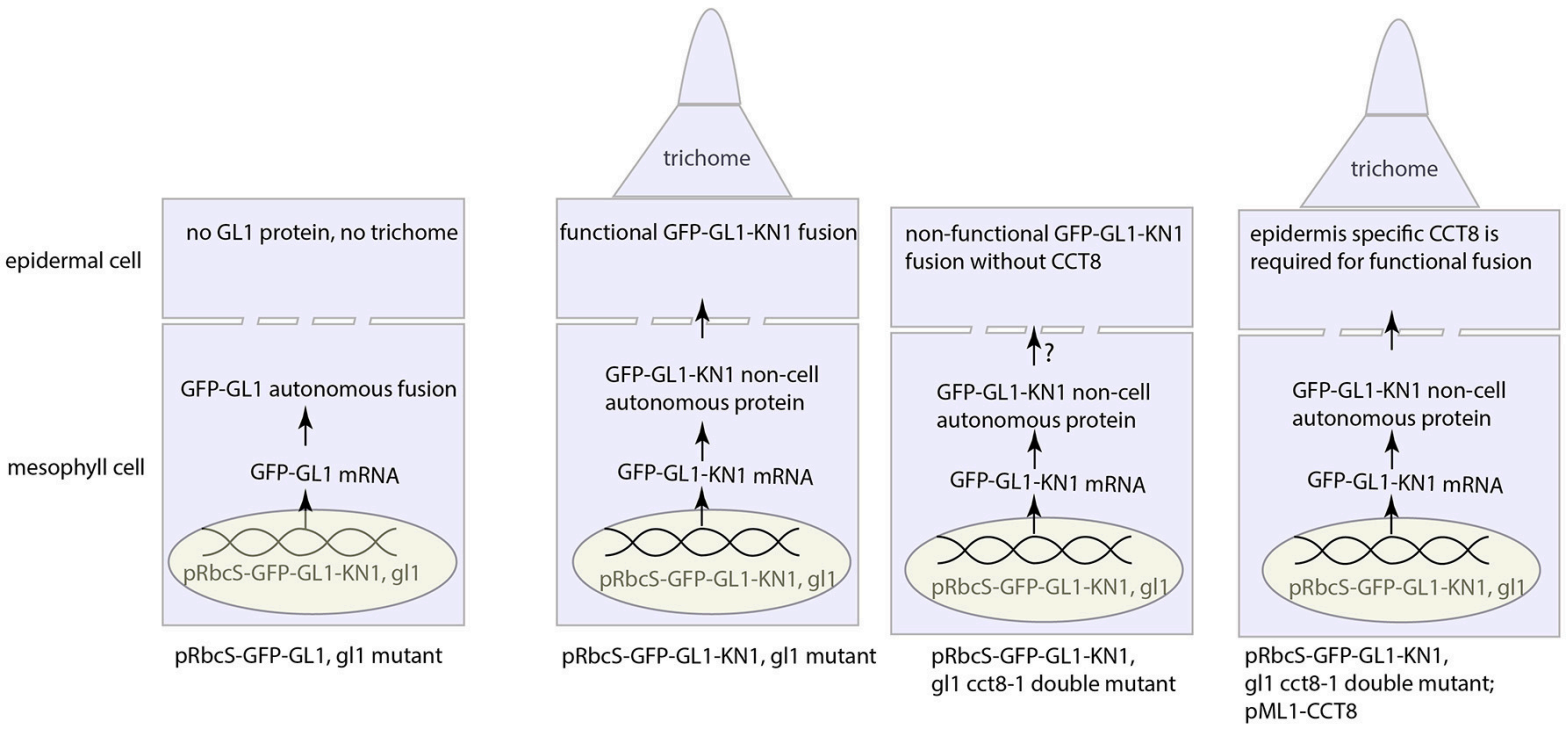
Intercellular KN1 movement through PD requires a post-movement refolding, which could result from ER stress. The non-cell autonomous feature of KN1C (trafficking domain of KN1) protein renders the GFP-GL1-KN1C fusion as a movement protein that moves from mesophyll cell to epidermal cell, whence the GL1 part in the fusion initiates the developmental program for trichome specification. Chaperonin CCT8 is required in the destination cell, in this case the epidermal cell, to refold the post-movement protein. This figure was drawn based on the description by Xu et al. (2011).

Another different approach involving the KN1 movement makes a surprising finding, where KN1 movement requires chaperone protein CCT8, a type-II chaperonin subunit (Xu et al., 2011) (Figure 4). Xu et al. (2011) further suggested that the whole chaperonin complex, known as TRiC/CCT complex, consisted of two rings with each having eight paralogous subunits (CCT1-8) (Horwich et al., 2007; Hartl et al., 2011), is required for unfolding and intercellular transport of KN1 protein. In addition to KN1 movement, movement of TRANSPARENT TESTA GLABROUS1 (TTG1) and, even external intruder such as oilseed rape mosaic virus (ORMV) also requires this complex (Xu et al., 2011; Fichtenbauer et al., 2012).

Most notably, CCT8 needs to be present in the destination cell, rather than the donor cell, to make the translocated GL1-KN1 fusion functional, however, this fusion, if natively expressed in the destination cell (*de novo* protein folding), is agnostic to CCT8 chaperonin (Xu et al., 2011). Therefore, re-folding after translocation through PD is critical for mobile protein to fulfill its function in new place. But, these observations, on the other hand, inevitably cast new thoughts on such questions as why chaperonin is needed for post-translocation re-folding, or whether there is any modification occurring to unfolded amino acid chain during passage through PD. Currently, there is no clue to these questions from literature, however, in the perspective of redox regulation on PD, it's tantalizing to conceive that the importance of chaperonin complex in PD-mediated protein movement is likely two-fold: (1) to unfold before transport and refold the impaired amino acid chain due to oxidative stress around PD after transport and (2) to protect mobile protein from redox fluctuation. Furthermore, protein folding/unfolding-induced endoplasmic reticulum (ER) stress, or more precisely the unfolded/misfolded protein-induced ER stress has been proposed to be a significant source of cellular ROS (Malhotra and Kaufman, 2007; Rutkowski and Kaufman, 2007; Santos et al., 2009), estimated to account for 25% of cellular ROS in the yeast *Saccharomyces cerevisiae* (Tu and Weissman, 2004). The similar mechanisms seem to operate in plants as well (Onda and Kawagoe, 2011; Aller and Meyer, 2013; Ozgur et al., 2014). Since ER sleeve runs through PD (Figure 2), ROS production from this process could be exploited to facilitate intercellular movement. Further studies need to be explored to reveal how chaperon protein help to move through PD channel and in what mode chaperon protein is incorporated with ROS to regulate intercellular communication.

## ROS are required to form intercellular/interorganelle bridge

### ROS induction of cellular membrane protrusions

Tunneling nanotubes (TNTs), usually considered as counterpart to PD, have recently been discovered in a variety of cell types (Rustom et al., 2004; Gerdes et al., 2007; Gurke et al., 2008; Rustom, 2009). These intercellular bridges that link mammalian cells are at play in transmitting a range of signals involved in developmental processes and spreading pathogens as well (Gerdes and Carvalho, 2008; Eugenin et al., 2009; Hase et al., 2009; Marzo et al., 2012; Abounit et al., 2016; Hashimoto et al., 2016; Victoria and Zurzolo, 2017). Thus, how TNTs are contributing to intercellular transport is becoming a very interesting, targeted topic since their discovery (Rustom et al., 2004). Accumulating evidence has shown that hydrogen peroxide can not only promote the formation of TNTs within the same cell type, e.g., between rat primary neurons, astrocytes (Zhu et al., 2005; Wang et al., 2011), and mouse CAD cells (Gousset et al., 2013), but also between heterozygous cell type, e.g., rat primary astrocytes and C6 glioma cells when co-cultured (Zhang and Zhang, 2015). Actually, more than hydrogen peroxide, oxidative stress induced by oxidant methylglyoxal and menadione can also promote TNTs formation in human peritoneal mesothelial cells (Ranzinger et al., 2014; Rustom, 2016) and rat astrocytes (Zhu et al., 2009) respectively. Moreover, a recent study has shown that movement of alpha-synuclein through TNTs is accompanied by increased ROS, suggesting that ROS could facilitate intercellular protein transport by increasing the number of TNTs (Abounit et al., 2016).

Notwithstanding these clear phenomena, the mechanisms underlying ROS-induced TNTs formation are just emerging from the perspective of their structural composition. Because TNTs are mainly actin-filled membrane protrusions (Austefjord et al., 2014), the inductive formation by ROS could be attributed to the effects on actin dynamics.

Indeed, cytoskeletal behaviors, under ROS stress, is widely shown to be altered (Raghu et al., 1986; Zhao and Davis, 1998; Gellert et al., 2015; Wilson and González-Billault, 2015; Xu et al., 2017), including actin polymerization, filament assembly, branching, cytoskeletal reorganization, and dynamic interactions with cytoskeletal regulators. These behavioral changes stem from either the direct oxidation of actin by ROS, or the ROS-initiated signaling, or both. Topologically exposed in the cytoplasm, some amino acid residues (e.g., Met 44, 47, 355, Cys 374) in actin and other cytoskeletal components are liable to oxidative attack (Lassing et al., 2007; Fedorova et al., 2010; Hung et al., 2011; Wilson et al., 2016), thereby inhibiting actin polymerization or association with other proteins (Dalle-Donne et al., 2002, 2003; Landino et al., 2002, 2007). These results are insightful; however, there are few reports showing the impairment of membrane protrusions are the direct result of actin oxidation. Part of this reason could be due to the powerful *in vivo* antioxidant system that can reverse the oxidized form. For example, under H_2_O_2_ treatment, actin can be kept in reduced form by thioredoxin-1 (Trx1) through its interaction with cysteine 62 of actin (Wang et al., 2010), thus antagonizing the direct oxidation. Similarly, for the oxidized Met, a methionine sulfoxide reductase SelR can reduce the oxidized Met 44 (Hung et al., 2013).

### Molecular signaling in TNTs formation

**Figure 5:**
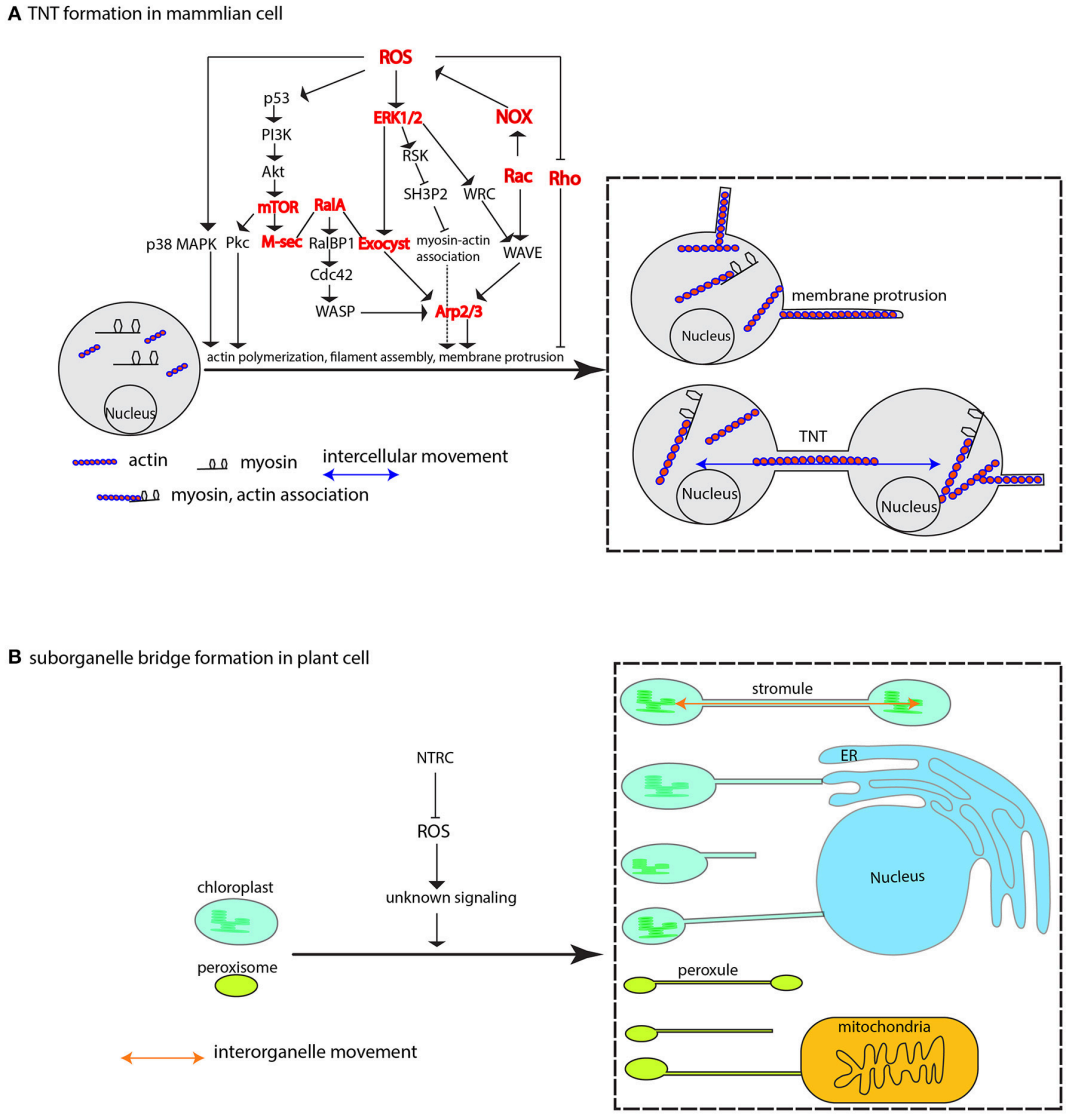
ROS-induced formation of membrane protrusion, intercellular and interorganelle bridge. **(A)** At physiologically higher ROS levels, TNTs are formed between cells via cytoskeleton-based (e.g., actin, myosin) membrane protrusion. ROS-induced diversified pathways including p38 MAPK, PI3k-Akt-mTOR signaling, ERK1/2 signaling, Rho GTPases family, and M-Sec-RalA-Exocyst complex, are shown to promote membrane protrusions mainly via Arp2/3-mediated actin cytoskeletal remodeling. The cooperative association between myosin and actin that is activated by ERK signaling plays an important role in mobilizing various related components and mediating membrane-cytoskeleton coordination. **(B)** In plant cells, the membrane-encircled organelles, e.g., the chloroplast and peroxisome, can form inter-organellar bridge between the same type of organelle (the stromule and peroxule) and different types of organelles under higher ROS condition. Repression of NTRC leads to increased stromules. The signaling mechanism is currently unknown. Arrows may not denote direct activation. Several key nodes in the signaling networks are colored in red, and Arp1/2 complex, Exocyst complex, NOX, ERK, p38 MAPK, Rac and Rho are also encoded in plant genomes. p38 MAPK, p38 mitogen-activated protein kinase; Pkc, protein Kinase C; PI3k, phosphatidylinositol-3-kinase; AKT, protein kinase B; mTOR, mammalian target of rapamycin; M-Sec, Myeloid and M cells-expressing Sec6 homolog, also known as TNF alpha-induced protein 2, or Primary response gene B94; RalA, Ras-related protein Ral-A; RalBP1, RalA-binding protein 1; Cdc42, Cell division control protein 42 homolog; WASP, the Wiskott–Aldrich Syndrome protein; ERK1/2, extracellular signal-regulated kinase 1; Exocyst, an octameric complex; RSK, ribosomal S6 kinase; SH3P2, Src homology-3 (SH3) domain-containing protein 2; Arp2/3, actin-related complex 2/3; WRC, WAVE2 Regulatory Complex; NOX, NADPH oxidase; WAVE, WASP family verprolin-homologous protein; NTRC, NADP-thioredoxin reductase C.

Apart from the direct interplay between ROS and antioxidant on the actin behavior, indirect cascade, or the ROS-initiated signaling could play important roles in dictating the actin-driven membrane protrusions. A pioneer work demonstrated that H_2_O_2_ induces TNTs-like protrusion formation by promoting actin polymerization and colocalzation of myosin Va and F-actin in the newly formed protrusions in primary rat astrocytes (Zhu et al., 2005). What's more, the property of astrocytes plasma membrane is also altered under ROS treatment, which seems not to be from the sheer oxidative damage on membrane, rather to be mediated by cytosolic phospholipase A_2_ (cPLA_2_) (Zhu et al., 2009). Further biochemical experiments suggested that H_2_O_2_-triggered phosphorylation of p38 MAPK and ERK1/2 (extracellular-signal-regulated kinase 1/2) is the key to protrusion formation as specific inhibitors of p38 MAPK and ERK can nearly abolish the H_2_O_2_-induced TNTs formation (Zhu et al., 2005, 2009). p38 MAPK and ERK1/2 are very import players in connecting various signaling pathways and involved in various fundamental processes (see reviews by Roux and Blenis, 2004; Shaul and Seger, 2007; Xu et al., 2014), so the question lingers about the MAPK cascade signaling specificity to TNTs formation. Fortunately, identification of the molecular players acting seemingly downstream of ERK1/2 could—at least partially—answer this question. A TNTs-like localized protein M-Sec induces *de novo* actin-containing protrusions through interaction with the activated small GTPase RalA and, mediation by RalA-exocyst complex (Sugihara et al., 2002; Hase et al., 2009). The exocyst complex has been shown to be directly involved in actin remodeling; one subunit of exocyst, Exo70, interacts with the Arp 2/3 complex (a key machinery for the generation of the filamentous actin network) to promote actin branching, resulting in membrane ruffling or protrusion (Zuo et al., 2006; Liu et al., 2009). A later discovery that ERK1/2 can directly phosphorylate the exocyst subunit Exo70 to enhance the complex assembly in response to EGF (epidermal growth factor) (Ren and Guo, 2012), established the link between ROS-initiated ERK1/2 signaling and M-Sec-RalA-Exocyst complex (Figure 5). Actually, in a similar situation to TNTs formation, ERK is also required for EGF-stimulated protrusion in human mammary epithelial cells. In this case, ERK promotes the lamellipodia protrusion by directly phosphorylating the WAVE2 Regulatory Complex (WRC), which then activates the Arp2/3 complex for actin assembly together with activated WAVE2 and Abi1 (activation by the ERK phosphorylation as well) (Mendoza et al., 2011). The mobilizing force to drive actin network remodeling may come from the myosin motor (Nambiar et al., 2010) that has been widely shown in the recent reports (Bishai et al., 2013; Lou et al., 2015; Yochelis et al., 2015; Saczko-Brack et al., 2016), so what factor, if there is any, is directing the force to this process? Interestingly, a recent report has pointed out that the ERK signaling could provide a clue in which ERK promotes lamellipodia protrusion by lifting the sequestration of myosin 1E by SH3P2 via phosphorylating its Ser 202, thus resulting in myosin-actin association at the leading edge of the human MKN1 tumor cell (Tanimura et al., 2016; Tanimura and Takeda, 2017).

Another study also showed that TNTs are induced by H_2_O_2_ in rat hippocampal astrocytes and neurons respectively. Genetic manipulation demonstrated that p53 is required for TNTs development, and that Akt is involved in TNTs induction by H_2_O_2_. Ly294002 and rapamycin, the inhibitor of PI3K and mTOR (the both are p53-responsive genes), respectively, can effectively quench the TNTs induction (Wang et al., 2011). Actually, an earlier report showed that mTOR together with RICTOR regulated actin organization by modulating the phosphorylation of Protein Kinase C α (PKCα) (Sarbassov et al., 2004). These collective data suggest the PI3K-Akt-mTOR signaling pathway is another key mechanism to regulate TNTs induction (Wang et al., 2011) through activating M-sec (Figure 5), or that the p38 MAPK and PI3K-Akt-mTOR might crosstalk to regulate their formation (Xu et al., 2014). Therefore, future studies in light of dissecting the relationship between the ROS-initiated p38, ERK, and PI3K-Akt-mTOR signaling are needed to advance our knowledge of TNTs formation under the direction of such complex signaling networks.

### Actin cytoskeleton-based cellular protrusions

Since actin cytoskeleton plays a crucial role during TNTs formation (Austefjord et al., 2014), it would be of interest to know whether ROS-triggered effects could be extrapolated to other actin-based membraneous protrusions, e.g., cytonemes (Ramírez-Weber and Kornberg, 1999; Hsiung et al., 2005), and actin-dependent outgrowth of neurite in neuron (da Silva and Dotti, 2002; Chia et al., 2016; Winans et al., 2016).

Remarkably, a physiological level of ROS sourced mainly from NOX and lipoxygenase is critical for neurite outgrowth; downregulation of ROS level with many different ROS scavengers caused the disassembly of actin cytoskeleton, resulting in reduced neuronal extension (Munnamalai and Suter, 2009). Different to the ROS signaling in TNTs outgrowth in the earlier reports by Zhu et al. (2005), Hase et al. (2009), and Wang et al. (2011), the signaling events for neuronal outgrowth are consisting of interactions between Rac and Rho GTPase-involved pathways (Figure 5) (Nimnual et al., 2003; Munnamalai and Suter, 2009; Chianale et al., 2010; Winans et al., 2016). Noteworthily, the Rac and Rho pathways are not tailored for neuronal outgrowth; they, together with Cdc42 and downstream componensts WAVE, WASP, and Arp2/3, are also required for TNTs biogenesis in macrophage (Hanna et al., 2017), and cytoplasmic protrusions during *Xenopus* embryonic development (Tahinci and Symes, 2003). Further studies on the *in vitro* neurite outgrowth have found that the protrusions, under certain circumstances, are also promoted by Akt-mTOR pathway (Jin et al., 2012) and p38 MAPK signaling pathway (Sarina et al., 2013), suggesting that neruronal outgrowth, similar to TNTs formation, can be achieved through diversified pathways. The wide availability of versatile signaling pathways may reflect the effective response of intercellular movement via formation of intercellular bridge to numerous environmental stimuli which more or less cause redox fluctuation. And eventually, the responsive node is converged on the actin cytoskeleton or other cytoskeletons possibly in accordance with the type of cellular protrusions (Figure 5).

Cytonemes are another type of intercellular bridges, similar to TNTs in shape, but distinct from TNTs in the contact sites—opened in the TNTs and closed in the cytonems. Since cytonemes are actin-based structure, it is plausible that cytonemes formation may be also subject to ROS regulation. This corollary does not guarantee an exact scenario, but a similar one. Actually, nitric oxide (NO), another type of reactive species (RNS, reactive nitrogen species), can drastically promote the formation of cytonemes on the surface of human neutrophil (Galkina et al., 2005) for targeting their prey at a distance (Galkina et al., 2009), and the inhibitor of nitric oxide synthase inhibit their extension without much impact on neutrophil spreading (Galkina et al., 2005). The exact inductive mechanisms are not fully addressed, however, based on the constituents of cytonemes, it's natural to speculate that NO may act through modulating cytoskeleton behaviors to extend cytomenes. Extensive studies have shown that nitric oxide synthase interacts with tubulin and actin cytoskeleton (Su et al., 2003, 2005; Kondrikov et al., 2006, 2010) and modulates their activity through tyrosine nitration and S-nitrosylation of target cytoskeletal proteins or cytoskeleton-associated proteins in both animals (Loesch et al., 1994; Aslan et al., 2003; Tedeschi et al., 2005; Thom et al., 2008; Zhang et al., 2015) and plants (Kasprowicz et al., 2009; Yemets et al., 2011; Yao et al., 2012; Blume et al., 2013; Rodríguez-Serrano et al., 2014). Thus, it's highly likely that these modifications on cytoskeleton, particularly the actin, could eventually impact on cytonemes formation.

However, this does not negate a possible role of ROS in cytonemes formation since several independent studies have shown that nitric oxide induces ROS production (Pieper et al., 1994; Lee et al., 2000; Patel et al., 2009). The NO-induced ROS can further trigger actin-based cellular protrusions, or alternatively, *in vivo* ROS generation could be achieved through the interaction of nitric oxide synthase with Rac protein (Jyoti et al., 2014), a key regulator of ROS production (Caron and Hall, 1998) (Figure 5). In fact, the NO-induced ROS pathway has been partially uncovered in another similar cellular protrusion in neutrophil. Neutrophil extracellular traps (NETs) are not surrounded by membranes, and appear thinner compared to cytonemes (Brinkmann et al., 2004). As in cytonems, NETs formation is induced with longer NO donor exposure, and this induction is dependent on the ROS generation from the mobilized NOX (Patel et al., 2010). Furthermore, genetic requirement of Rac and NOX for NETs formation reinforces the ROS part in the NETs protruding process (Lim et al., 2011; Stojkov et al., 2017).

The Rac and NOX components are also involved in the neutrophil chemotaxis by affecting actin behaviors. For example, NOX-dependent ROS negatively regulate actin polymerization via actin glutathionylation, and disruption of antioxidant enzyme glutaredoxin 1 (Grx1) leads to attenuated actin polymerization (Sakai et al., 2012). In mouse bone marrow neutrophil, Rac knockout significantly reduced superoxide level and neutrophil cell polarization toward a chemoattractant gradient (Roberts et al., 1999). Taken together, cytonemes formation, presumably via Rac-dependent actin cytoskeletal remodeling (Mitchell et al., 2008), might share the similar mechanisms to that underlying the TNTs and neuronal outgrowth (Figure 5). But before leaping to this conclusion, further study needs to be carried out to examine the relationship between RNS and ROS and, the crosstalk in signaling the membrane protruding process.

### Membrane protrusions in plant cells

As discussed above, the ROS-induced membrane protrusion via actin remodeling is widely seen in many different mammalian cell types. So, would an analogous situation occur in plants as well? Interestingly, rather than displaying the increased simple PD between cells, the *ise1* mutant with elevated ROS level as discussed earlier is developing more complex PD such as the twinned and branched PD (Burch-Smith and Zambryski, 2010), whose formation requires membrane insertion into pre-existing simple PD (Faulkner et al., 2008; Burch-Smith and Zambryski, 2012). Nevertheless, this process is much more complicated than the intercellular bridge formation between mammalian cells as it has to deal with cell wall remodeling, e.g., cell wall thinning (Ehlers and Kollmann, 2001; Ehlers and Westerloh, 2013). Thus, membrane protrusion induced by oxidative stress during complex PD formation may not typically exemplify the role of oxidative stress in this process, which is indeed the case in a new study on the formation of complex PD in *Arabidopsis* leaf epidermis (Fitzgibbon et al., 2013). Other relative simple membrane structure, without wall sophistication, could be more ideal to reflect the role of ROS in membrane protrusion in plants (see below for further discussion).

### ROS induction of interorganelle structure: stromule and peroxule

Some specialized membrane contact sites, or organelle extensions such as stromule, peroxule, matrixule (Mathur et al., 2012; Pérez-Sancho et al., 2016)—evidently without the complication of cell wall remodeling—present ideal platforms to address the mechanisms of membrane extrusions. Stromules are stroma-filled tubules that may, *in vivo*, extend—analogous to TNTs—along actin microfilaments, myosin and ER (Kwok and Hanson, 2003, 2004; Gunning, 2005; Natesan et al., 2009; Sattarzadeh et al., 2009; Schattat et al., 2011). Stromule formation is known to be induced by different kinds of stresses including hydrogen peroxide (Gray et al., 2012). A recent study further elaborated this association. Brunkard et al. (2015) found that formation of stromule from photosynthetic chloroplast, not non-photosynthetic plastid or leucoplast, can be boosted by the chemicals that specifically elicit ROS production in the chloroplast. This result is further genetically reinforced by virus-induced gene silencing of the chloroplast-localized NADP-thioredoxin reductase C (NTRC), which serves as reductant to reduce hydrogen peroxide (Brunkard et al., 2015); more than doubled stromule frequency in the *NTRC* silencing leaf of *N. benthamiana* is, therefore, presumably construed as the consequences of the ROS accumulation in the chloroplast (Brunkard et al., 2015; Hanson, 2015) (Figure 5). Since *in vitro* isolated chloroplast extends stromule independently of cytoplasmic niche (Brunkard et al., 2015; Ho and Theg, 2016), it would be very intriguing to see the ROS effect is still viable on the *in vitro* stromule formation, which could resolve the questions as to whether and/or how ROS effect is working through cytoplasmic niche.

A similar morphing response to ROS stress was also discovered in peroxisome's extension (Sinclair et al., 2009; Rodríguez-Serrano et al., 2016), namely the peroxule which is coined after stromule as these membrane protrusions are morphologically similar (Scott et al., 2007). The response is very rapid, and peroxule extension occurs within seconds of exposure to H_2_O_2_ and ·OH radicals (Sinclair et al., 2009). Similarly, high light stress, a condition in which cytosolic H_2_O_2_ elevation is induced, can bridge peroxisome-mitochondria contacts via peroxule (Figure 5). In addition, an irrelevant mutant to peroxule formation, the *anisotropy1* defective in cellulose synthase (CesA) gene displays elevated internal H_2_O_2_ level, and at the same time has higher peroxule frequency compared to wild type plant (Jaipargas et al., 2016). Noteworthily, other ROS like singlet oxygen and superoxide seem not to instigate peroxule growth (Sinclair et al., 2009), suggesting peroxule formation may rely on specific ROS-dependent signaling pathway (Sinclair et al., 2009). The proposition is recently revealed that quick peroxule formation is achieved through NOX-generated ROS-mediated PEX11a expression—at least in the case of Cd stressing situation (Rodríguez-Serrano et al., 2016).

All of the accumulating evidence highlights a conservative phenomenon in which the membrane protrusions are subject to oxidative change, particularly H_2_O_2_ fluctuation, either internally or externally. In mammalian cells, the ROS-initiated signaling will eventually converge on the regulation of cytoskeleton that underpins the cellular protrusion. The key players in the signaling pathways that direct membrane protrusions are largely found in plant cells, e.g., the Arg2/3 complex (Yanagisawa et al., 2013), Rac and Rho family (Hassanain et al., 2000; Nagawa et al., 2010; Kawano et al., 2014), exocyst complex (Zhang et al., 2010), NOX (Wang et al., 2016), ERK (Cvetkovska et al., 2005; Furukawa et al., 2012), and p38 MAPK (Liu and He, 2017), implying that the signaling pathways in animals have meandered into the networks controlling the cytoskeleton remodeling in plant cells; an example is the exocyst complex is required for pollen tube growth (Bloch et al., 2016), for which extensive membrane dynamics and cytoskeletal remodeling are taking place at the tip of pollen tube (Cheung and Wu, 2008; Kroeger and Geitmann, 2012; Qu et al., 2015). Although the exact mechanism for organellar extension is still under investigation, it's not hard to conceive that characterization of the structural compositions of these extensions including the membrane skeleton associated with them would represent a solid, but very challenging step toward the comprehension of such complicated process given the small size of these extensions. ROS-initiated signaling pathways can be further elaborated with the insights gained from TNTs studies.

## Increased or decreased transport via PD: amount talks

ROS level has to be kept in a certain range to maintain a healthy cellular status, in which the intercellular movement alters with ROS concentration accordingly. As shown by Rutschow et al. (2011) and Liang et al. (2014), an uptick in hydrogen peroxide supplement can increase intercellular movement, but this effect can be reversed if the extra concentration has reached a very higher level of more than 3 or 6 mM. This phenomenon as well as the contradicted results from *gat1* and *ise1* mutant needs a further explanation although it might be associated with subcellular redox as discussed in previous section. Another possible way to work around this issue could borrow insights from recent work on pollen tube rupture and pathogen penetration.

### Oxidative burst and cell wall disconnection/dissolution

**Figure 6:**
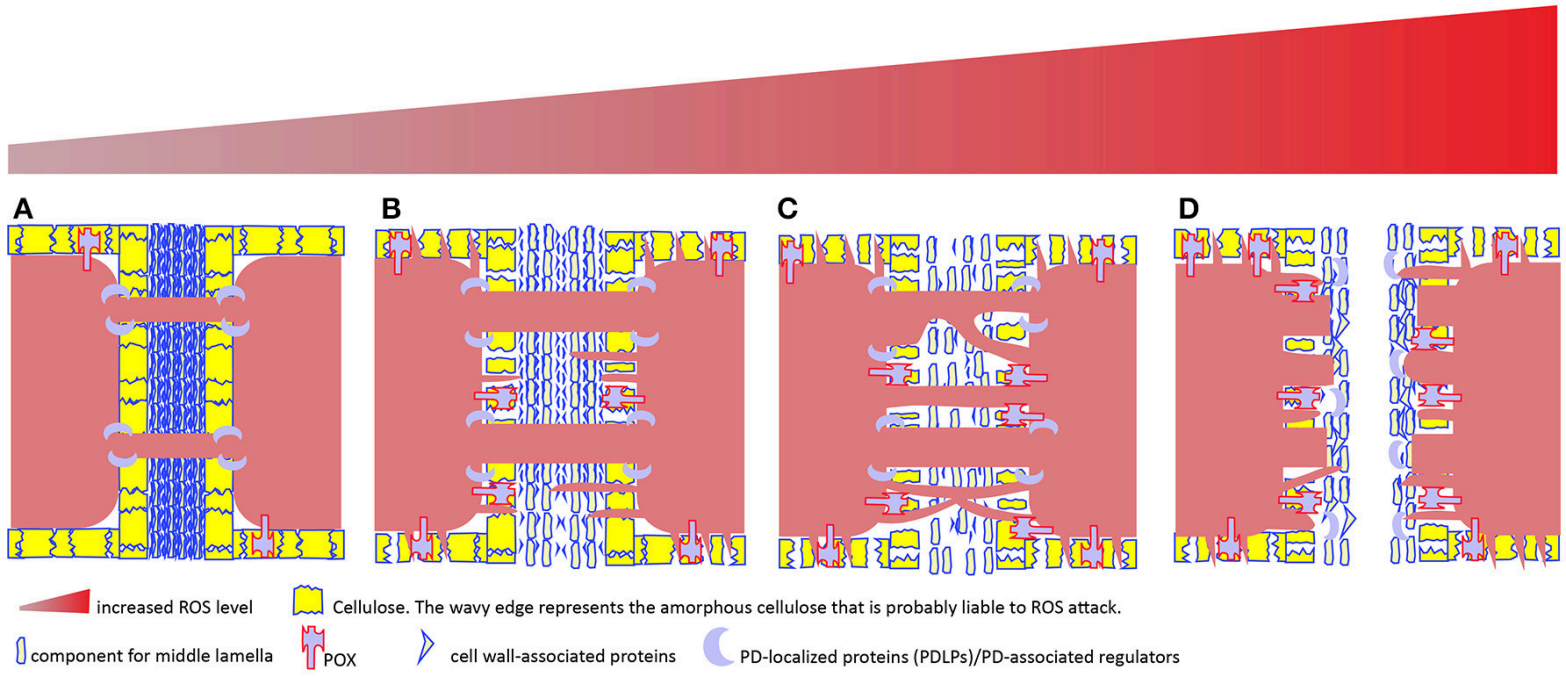
A tentative model to depict the relationship among cell wall remodeling, membrane protrusion, complex PD formation and increased ROS level in plant cells. Plasmodesmata (PD)-mediated intercellular transport is usually regulated by PD-localized proteins (PDLPs) and other PD-associated regulators (e.g., callose synthase, glucanase), and involves no cell wall remodeling **(A)**. In apoplast, ROS-generating enzymes including type III peroxidase (POX) and other cell wall-localized oxidases drive cell wall remodeling. Apoplastic H_2_O_2_ increment and subsequent conversion into hydroxyl radical from H_2_O_2_ leads to cell wall loosening via oxidative scission of cell wall polysaccharides and ROS-activating enzymatic breakdown of cell wall-associated proteins. PD embedded in the loosened cell wall expands **(B)**, resulting in increased intercellular exchange. In the symplast, H_2_O_2_-induced protruding membrane may cross the loosened cell wall to form novel PD or fuse with existing PD to form complex PD **(C)**. However, too high ROS cause either cell death (apoptosis) or middle lamella dissolution, resulting in the physical cut-off between cells **(D)**.

A ROS burst usually occurs under biotic/abiotic stress in order to kill invaded pathogen or trigger hyper-sensitive cell death to protect plants from detrimental effects induced by stressors. Such occurrence is a dangerous theme to plant itself, so employment of high ROS for plant normal development is thought to be rare. However, recent study reported that pollen release from the tube into the female gametophyte during reproductive process requires the cell wall rupture of pollen tube, which is achieved via a NOX-mediated ROS burst (Duan et al., 2014) as it happens during root hair growth (Foreman et al., 2003; Orman-Ligeza et al., 2016). Moreover, this process can be reconstituted *in vitro* by providing exogenous 1 mM H_2_O_2_ (Duan et al., 2014), clearly demonstrating oxidative burst is required for pollen tube rupture. In a broader biological sense, this scenario has reminded us that oxidative burst plays critical roles in cell wall dissolution, as has been evidenced in abscission process in various species (Sakamoto et al., 2008a,b; Yang et al., 2015; Liao et al., 2016). What's more, ROS burst at the site of abscission is most possibly mediated by peroxidase (Poovaiah, 1973; Hall and Sexton, 1974; Henry, 1979; McManus, 1994). A subclass of anatomically distinct cells—smaller size and denser cytoplasm compared to neighboring cells—makes up of the abscission zone, from which cell wall loosening and eventual dissolution will occur under the control of various cell wall remodelers (Roberts et al., 2002; Kim et al., 2015; Merelo et al., 2017; González-Carranza et al., 2018). To be more specific, dissolution is most likely happening in the lamella zone of cell wall (Figure 6) (Agustí et al., 2012; Yamada et al., 2015).

One obvious consequence from abscission is the complete physical cut-off from intercellular transport, although questions remain as to at which stage and in what way the PD permeability is blocked during formation of abscission layer, given the impact of ROS burst and vigorous cell wall remodeling on PD structure and/or permeability.

Interestingly, a recent study on oil palm fruit abscission zone shows that abscission zone is multi-cell layered structure and PD is frequently observed within the layer, but less frequently observed between layers (Roongsattham et al., 2016), suggesting that inter-layer communication via PD is becoming less likely prior to separation. With progression toward abscission, the PD size within layer is enlarged (Roongsattham et al., 2016), fusing membrane vesicles, highly branched PD and its associated cavities in the vicinity of middle lamella are frequently observed (Osborne and Sargent, 1976; Henry, 1979; Bar-Dror et al., 2011; Roongsattham et al., 2016). The physiological function of these branched PD in the abscission layer is yet known, but clues from the presence of branched PD in the trichome-epidermal boundary (Faulkner et al., 2008) where there is only unidirectional transport (Christensen et al., 2009) (Figure 1) and also in the cells undergoing sink-source transition that allows carbon export (Volk et al., 1996; Oparka et al., 1999) suggest that the branched PD in the abscission zone may be involved in the unidirectional transport between the separated tissue and the main body. Alternatively, they are simply results of the vigorous cell wall remodeling, or formed from the fusions of existing simple PD due to ROS burst which would also boost the polarization of membranous system, such as the TNTs formation or stromule, peroxule formation as discussed previously (Figure 6).

### Callose, PD transport, and pathogen penetration

Decades of research on pathogen-plant interaction have established that cell wall barrier, e.g., cell wall appositions known as papillae, at the site of infection can be formed to constitute a first physical front line against intruder penetration (Aist, 1976), and later that strengthening of this specialized structure is proposed to be driven by ROS burst during infectious attack (Hückelhoven et al., 1999; Grant and Loake, 2000; Torres et al., 2006) as well as iron deposition (Liu et al., 2007). Callose, the most abundant constituent of papillae (Aist, 1976; Schulze-Lefert, 2004; Bellincampi et al., 2014; Voigt, 2014), is considered to assemble quickly at attack site, thereby forming a barrier to resist intruder's penetration. However, this prevailing view does not withstand from two independent genetic studies in which loss of function of *GLS5*/*PMR4* gene coding for a GLUCAN SYNTHASE, indeed, leads to lack of callose deposition at papilla, but paradoxically results in the effective growth cessation of powdery mildew (Jacobs et al., 2003; Nishimura et al., 2003). And more than that, overexpression of the same gene in *Arabidopsis*, leading to enlarged callose deposits and focal accumulation at sites of attempted fungal penetration, again, jibed with the traditional wisdom (Ellinger et al., 2013).

Therein lies the seemingly irreconcilable contradiction and the role of callose in defending fungal penetration is becoming less conclusive. The structural components of papillae has not been fully understood, therefore, the possibility remains that other factors, beyond the sole function of callose, may account for the discrepancy. By examining the availability of various polysaccharides in barley papillae against *Blumeria graminis f*. sp. *hordei*, a new study combining immunofluorescence with immunogold-labeling methods reveals that an effective papilla is a multilayered structure, with the inner core consisting of callose and arabinoxylan and the outer layer containing arabinoxylan and cellulose (Chowdhury et al., 2014). Early results showed that inhibition of H_2_O_2_ production by secreted catalase from *B. graminis f*. sp. *hordei* during penetration might interfere with penetration resistance (Zhang et al., 2004), and that inhibition of a POX-mediated ROS burst via direct inactivate of peroxidase activity by fungal effector Pep1 also suppressed penetration resistance (Hemetsberger et al., 2012). These findings allow the proposal of an integrated model in which POX-dependent ROS accumulation together with the multilayered cell wall organization shapes up a resistance barrier to hyphae penetration (Hückelhoven, 2014).

Given the facts that callose homeostasis at the neck region of PD is one of the key mechanisms regulating permeability (Zavaliev et al., 2011; De Storme and Geelen, 2014; Knox and Benitez-Alfonso, 2014) and that PD is exploited by fungal hyphae for spread, at least in the case of rice blast fungus (Kankanala et al., 2007), and also resided with various sensing proteins to detect intruder's presence, thus to trigger its closure (Lee et al., 2011; Faulkner et al., 2013; Caillaud et al., 2014), one may speculate that similar scenario is likely to occur for PD permeability. That is, PD permeability, analogous to hyphae growth through cell wall and/or PD, is regulated by more than callose; cell wall components and how they are organized, and POX-mediated ROS level are acting in concert to determine PD permeability (Figures 2, 6).

## An evolutionary logic to ROS-regulated intercellular movement

ROS-controlled biomolecule movement and intercellular/interorganellar bridge formation, which can facilitate intercellular transfer, in both plants and animals suggest there could be existence of some evolutionary sense that needs to be comprehended. Actually, intercellular communication, as has been proposed from an evolutionary standpoint, is one of the key fundamental traits that are required to be developed for the successful evolution of complex multicellular organisms (Knoll, 2011; Niklas and Newman, 2013). Due to the essential notion in this review expressing that intercellular movement of many types of molecules is controlled by ROS, it would be justifiable to link the role of ROS in intercellular movement to multicellularization. This association is further bolstered by the findings that assigned ROS generation to multicellular organ formation.

Take NOX as an example. A study with *Dictyostelium* directly defined superoxide as the key signal in its transition from the single to multicellular phase (Bloomfield and Pears, 2003). Without superoxide, the multicellular aggregates simply do not form. The authors further indicate that the source of ROS could result from NOX activity. The ensuing study from independent group demonstrated that insertional disruption in several *NOX* gene of *Dictyostelium* indeed gave out defects in the formation of mature fruiting bodies—the multicellular structure (Lardy et al., 2005), genetically consolidating the role of superoxide in multicellular formation.

Actually, NOX enzyme, the ROS generating machinery exists in all multicellular organisms, and is absent from majority of unicellular organisms (Lalucque and Silar, 2003; Hervé et al., 2006; Nguyen et al., 2017). The NOX family has been proposed to be important innovations for the evolution of multicellularity (Blackstone, 2000). Although unicellular fungus such as *S. cerevisiae* does harbor NOX gene (Rinnerthaler et al., 2012), this does not necessarily argue against the role of ROS-generating machinery in multicellularity as *S. cerevisiae* can undergo cellular fusion to form a diploid zygote which needs intercellular signaling (Merlini et al., 2013). What's more, several oxidants including H_2_O_2_ can activate mating-responsive genes (Staleva et al., 2004). For complex fungi, NOX deletion does not affect hyphal growth or asexual development, but impairs formation of fruiting body in fungi *Aspergillus nidulans* and *Podospora anserina* (Lara-Ortíz et al., 2003; Malagnac et al., 2004), strongly suggesting the presence of ROS-generating machinery is highly correlated to multicellular structure.

As for the existence of NOX family in unicellular algal organisms (Hervé et al., 2006; Anderson et al., 2011; Chang et al., 2016), studies so far have not yet determined the biological role of NOX in unicellular algae. However, it's feasible to speculate that the NOX gene could be, as has been reviewed in the section about stromule formation, used to generate ROS to facilitate inter-organelle communication in these algae. Further experiment is needed to clarify this assumption.

NOX is ROS-generating machinery, and its activity is required, at least in fungi, for multicellular formation, but the question remains how the activity is regulated in light of multicellularization. Until recently, an excellent study in *C. elegans* has uncovered a new regulatory mechanism involving tetraspanins (Moribe et al., 2012). Tetraspanins are another well-known multicellularity-associated gene family, which is first innovated in multicellular organisms (Berditchevski, 2001; Huang et al., 2005). Its main role in multicellularization is to facilitate cell adhesion and cell-cell communication (Wang et al., 2012; Charrin et al., 2014). Moribe et al. (2012) showed that null mutation of a tetraspanin gene *Tsp-15* causes the worm to be very short and croissant-like phenotype, and leads to embryonic lethality. Genetic screens together with Co-IP and cell-fusion technique identified a concurrent genetic and biochemical pathway in which H_2_O_2_ production is dependent on Tetraspanin (TSP-15)-activated NADPH dual oxidase (BLI-3)-maturation factor(DOXA-1) complex (Moribe et al., 2012). The downstream signaling pathway involves peroxidase MLT-7, initiating the extracellular crosslinking process (Thein et al., 2009; Moribe et al., 2012). Although such clear insights are lacking in mammals and plants, emerging work in fungal genetic studies has shown that ROS production and Tetraspanins appear to work concurrently to establish cell-cell contact during fungi-host interaction (Moribe and Mekada, 2013). Deletion of either tetraspanin gene *PLS1* (Clergeot et al., 2001; Gourgues et al., 2004; Veneault-Fourrey et al., 2005) or NOX genes *nox1/nox2* (Egan et al., 2007), or both genes in *P. anserina* (Lambou et al., 2008) and *Botrytis cinerea* (Siegmund et al., 2013) renders the fungus “punchless,” a condition in which the fungi have defects in penetration. Noteworthily, involvement of tetraspanins and NOX in pathogen-host relationship may not specifically contribute to the pathogenicity, rather hints at an evolutionary strategy to establish interface, thereby enabling transport of nutrients and signaling molecules between fungal and host cells (Bonfante and Genre, 2010). Such is the case with symbiotic interface. NOX-generated ROS in the extracellular matrix is required for fungus *Epichloë festucaeis* to establish mutualistic association with perennial ryegrass *Lolium perenne* (Tanaka et al., 2006). Further studies with other symbiotic models could reveal more common grounds of mutual interactions, such as ROS production and its relationship with cell wall remodeling (Lionetti and Métraux, 2014; Martin et al., 2017).

For now, we have learned intercellular transport is the key underpinning to multicellularization and other forms of cellular interaction, e.g., the pathogenic and mutualistic interface, all of whom needs ROS to be involved. Strikingly, geological and archeological records together with phylogenetic analysis have shown that rise and fall of atmospheric oxygen is highly correlated with multicellular emergence and disappearance (Hedges et al., 2004; El Albani et al., 2010, 2014; Schirrmeister et al., 2013). Thus, evolutionary tendency to favor ROS utilization in transport may be incorporated into an evolutionary logic in which oxygen and its associated reactive species contribute to drive the evolution of multicellular life by aiding intercellular transport.

## Conclusions

By summarizing the data from across the artificial dye movement, protein movement, gene silencing movement, and intercellular bridge formation we can readily add the role of ROS in facilitating intercellular transport into their signaling repertoire. The ROS effects on membrane protrusions are widely conserved in many different types of animal cells, although diversified signaling networks are present accordingly in different occasions. Key pathways and players involved in membrane protrusions are well-conserved in plants and animals. Superimposed on the plasma membrane, the cell wall in plants poses another challenge to intercellular communication. Remarkably, ROS are still needed for cell wall remodeling. Distinct from NOX that resides on cell membrane and is required for membrane protrusions in animals, the apoplatic player POX that is unique to land plants and located on cell walls (Passardi et al., 2004a,b), is evolved for cell wall remodeling during the evolution of land plants. Convincingly, this role for ROS has actually found its evolutionary logic deeply rooted in the evolution of multicellularity as intercellular communication is one of the key prerequisite for evolving a multicellular organism. Therefore, it is not unintelligible to learn that ROS are widely associated with lots of, if not all of the, physiological processes. If there exists a mechanistic commonality among different physiological processes, then plasmodesmatal path or intercellular bridges could be a candidate hub that converges and dispatches the signaling molecules from various developmental and physiological processes for a coordinated response. This would encounter another inevitable question which is how ROS-enabled biomolecule movement is engaging with intercellular gating/selection. So far, we have no candidate answer. With technique advancement in *in situ* ROS detection, characterization, and resolution of signaling networks, more hidden features are expected to be unearthed in this perplexing but also intriguing area.

## Author contributions

The author confirms being the sole contributor of this work and approved it for publication.

### Conflict of interest statement

The author declares that the research was conducted in the absence of any commercial or financial relationships that could be construed as a potential conflict of interest.
